# Three-Dimensional Printed MXene@PANI Hierarchical Architecture for High-Performance Micro-Supercapacitors

**DOI:** 10.3390/ma18102277

**Published:** 2025-05-14

**Authors:** Anyi Zhang, Yiming Wang, Haidong Yu, Yabin Zhang

**Affiliations:** 1 Guangxi Key Laboratory of Processing for Non-Ferrous Metals and Featured Materials, MOE Key Laboratory of New Processing Technology for Non-Ferrous Metals and Materials, School of Resources, Environment and Materials, Guangxi University, Nanning 530004, China; ayzhang0329@163.com (A.Z.); yimingwang001@163.com (Y.W.); 2 School of Chemistry and Chemical Engineering, Guangxi University, Nanning 530004, China

**Keywords:** 3D printing, electrochemical performance, micro-supercapacitor, MXenes, polyaniline

## Abstract

The advent of the Internet of Things has boosted portable and wearable miniature electronics, especially micro-supercapacitors (MSCs) with excellent integrated performance as well as high-power density and a long lifetime. However, the rational design of electrode material formulations and the construction of three-dimensional (3D) structured electrodes with scalable and cost-effective fabrication remains an arduous task for improving the energy density of MSCs to meet all industrial sector requirements, such as the mass-production of microscale structures, a lasting power supply, and safety. To address these challenges, combining the respective capacitance merits of MXenes and polyaniline (PANI), we propose a constructing strategy for the preparation of a 3D MXene@PANI hierarchical architecture consisting of one-dimensional (1D) PANI nanofibers grown on two-dimensional (2D) Ti_3_C_2_ MXene nanosheets via extrusion-based 3D printing. Such a 3D architecture not only achieves a high loading mass of MSC electrodes prior to conventional planar MSCs for abundant active site exposure, but it also overcomes the restacking of MXene nanosheets accounting for sluggish ionic kinetics. These features enable the resulting MSCs to deliver excellent electrochemical properties, including a high volumetric capacitance of 1638.3 mF/cm^3^ and volumetric energy density of 328.2 mWh/cm^3^. This power supply ability is further demonstrated by lighting up a blue bulb or powering an electronic thermometer. This study provides a promising design strategy of the architecture of MXene@PANI composites for high-performance MSCs with 3D printing technology.

## 1. Introduction

With the advent of the intelligence era and rise of the Internet of Things (IoT), the growing demand for portable and wearable electronics has stimulated the development of advanced microscale energy storage devices, which have emerged as a promising energy provider for micro-applications due to their integrated performance [1,2]. Usually, they include two promising micro-batteries (MBs) and micro-supercapacitors (MSCs), which maintain microdevices distributed in the IoT for long-term operation. Of these microscale energy storage devices, MBs typically exhibit a high energy density of ≈1 mWh/cm^2^ but low power density and poor cycling performance caused by a slow and diffusion-controlled electrochemical process [3], whereas MSCs are recognized for their outstanding high power density, long operating life, fast charge–discharge rate, good mechanical robustness, easy maintenance, and excellent security, offering a promising on-chip energy storage solution. However, relatively low energy density (usually 0.01 mWh/cm^3^) often impedes their wide practical implementation [4]. It has been demonstrated that the electrochemical performance of MSCs is closely dependent on the electrode active materials and designed structure [5]. Consequently, the selection of electrode materials with high conductivity and large charge-storage capacity is pivotal for achieving high-performance MSCs.

MXenes are an emerging family of 2D layered transition metal carbides, nitrides, and/or carbon nitrides. The general formula of MXenes is M_n+1_X_n_T_x_, prepared by selectively removing the “A” layer (typically Al or V) from the MAX phase, where “M” represents transition metals (e.g., Ti or V); “X” represents the C, N, or CN, T_x_ stands for surface terminal groups (e.g., -F, -O, or -OH); and “n” is usually an integer between 1 and 3. MXenes have demonstrated encouraging prospects for energy density improvements in manufactured MSCs as capacitor-type electrode materials due to their exceptional metallic conductivity, abundant surface functional groups, high electrochemical activity, large specific surface area, and good mechanical properties [6,7]. However, dense restacking of 2D MXene nanosheets, due to van der Waals forces, results in poor ion diffusion kinetics, sloping charge/discharge profiles, and relatively low areal energy density. Moreover, exfoliated Ti_3_C_2_T_x_ is susceptible to hydrolysis in a water-based solution or at high temperature, leading to inherent instability of the structural components [8]. Hence, numerous endeavors have been undertaken to address these issues for the electrochemical performance enhancement of MXene-based microelectrodes. Considering the performance dependence on the material structure and components, on the one hand, MXenes are modified by tailoring the pore architecture, interlayer spacing, surface groups, and heteroatom doping. On the other hand, MXenes are functionalized and hybridized with other advanced active materials, such as carbon materials, metal oxides/sulfides, and conducting polymers [9,10,11]. In view of the synergy of multiple components, MXene-based compounds with a designed structure and hybrid component have recently been proposed to enhance the performance of MSCs [12,13]. It has been demonstrated that the incorporation of redox-active 1D nanofibers into 2D layered nanomaterials is an effective strategy to significantly enhance the MXene performance [14]. Polyaniline (PANI), a typical conducting polymer with good pseudo-capacitance, has proven effective for improving electrochemical performance because of its excellent energy storage ability, adjustable physicochemical properties, and facile synthesis of 1D structures [15,16,17]. The combination of PANI with MXenes to prepare composite electrode materials is anticipated to improve Faradaic pseudo-capacitance and electric double-layer capacitance. Moreover, abundant electronegative sites on the surface of MXenes can induce the in situ polymerization and growth of polyaniline as an antioxidant layer, thereby effectively surmounting the easy oxidation of MXenes and inhibiting the self-stacking of MXenes. Up to now, there are some works on MXene@PANI composites for SCs through diverse hybridization, but with less attention paid to the intricate structure design of these composites [18,19,20,21]. This is especially true when the size of the electrodes is scaled down to the millimeter range with the low-resolution, conventional planar structures with low mass loading result in low energy density, making it difficult to meet the application requirements.

Given the contribution of the 3D electrode structure’s establishment to high mass loading, much research has begun to build MXene/PANI composites with a designed hierarchical architecture to facilitate an interfacial reaction between the electrode and electrolyte [22,23,24]. Unfortunately, constructing MXene/PANI electrodes with complex 3D structures remains an arduous task for high-performance MSCs. Traditional MXene/PANI preparation methods, including spray-masking [25], electrochemical deposition [26], sputtering [27], laser-scribing [28], and photolithography [29], are either difficult for manufacturing 3D microelectrodes efficiently and stably or complex and costly. As an emerging, cost-effective, and multifunctional technology, 3D printing technology could fabricate pre-designed geometric shapes with enhanced fidelity by layer-by-layer stacking [30]. Microelectrodes with complex 3D structures and high active substance loading have been achieved in a limited area, finally effectively enhancing the electrochemical performance of customizable MSCs [31]. Consequently, it is crucial to develop highly effective and efficient strategies to manufacture MXene@PANI composites with a 3D hierarchical structure and assemble them into 3D thick microelectrodes for high-performance MSCs.

To this end, combining the respective capacitance merits of MXenes and PANI, we proposed a constructing strategy for the 3D MXene@PANI hierarchical architecture consisting of 1D PANI nanofibers grown on the surface of 2D Ti_3_C_2_T_x_ MXene nanosheets via a low-temperature dilute solution oxidative polymerization method. The incorporation of PANI nanofibers not only prevents the restacking of MXene nanosheets accounting for sluggish ionic kinetics but also improves stability and increases its redox sites for fast ion transport and electron transfer, contributing to excellent capacitance performance. After tuning the rheological properties, the MXene@PANI ink was obtained and then printed into 3D interdigital microelectrodes. The thickness of the 3D interdigital microelectrodes ranged from 0.6 mm to 4.1 mm. When they were encapsulated into MSCs with sealed films and gel electrolytes, the resultant MXene@PANI MSCs delivered a high volumetric capacitance of 1638.3 mF/cm^3^, volumetric energy density of 328.2 mWh/cm^3^, and good stability after 10,000 charging/discharging cycles, which is further demonstrated by powering a real bulb or electronic meter. This work opens a new avenue for designing the 3D composite electrodes of MSCs.

## 2. Material and Methods

### 2.1. Materials

Ti_3_AlC_2_ MAX was purchased from Shandong Xiyan MXene New Material Shop, Heze China. LiF, aniline, ammonium persulfate (APS) [(NH_4_)_2_S_2_O_8_], PVA, and Na_2_SO_4_ were purchased from Shanghai Aladdin Biochemical Technology Co., Ltd., Shanghai, China. HCl and HClO_4_ were provided by Nanning Lantian Experimental Co., Ltd., Nanning, China.

### 2.2. Synthesis of Single-Layer Ti_3_C_2_T_x_ Nanosheets

In a PTFE beaker, 1 g of LiF was dissolved in 20 mL of 4 M HCl, 1 g of Ti_3_AlC_2_ MAX powder was slowly added in a flowing-water form during the stirring process, and the etched multilayer Ti_3_C_2_ (m-Ti_3_C_2_) aqueous solution was obtained by fully stirring the reaction at 35 °C for 24 h. Subsequently, deionized water was added to the m-Ti_3_C_2_ aqueous solution and washed several times through a low-temperature high-speed centrifuge (6000 rpm/min 5 min each time) until the pH of the supernatant was neutral. After pouring out the supernatant, the m-Ti_3_C_2_ precipitate at the bottom was dissolved in deionized water and placed in an ultrasonic cleaner to fully disperse for two hours, and a uniformly dispersed single-layer Ti_3_C_2_ (s-Ti_3_C_2_) nanosheet solution was obtained. Finally, the s-Ti_3_C_2_ nanosheet solution was freeze-dried to obtain fluffy aerosol-shaped s-Ti_3_C_2_ foam.

### 2.3. Synthesis of MXene@PANI Composites

Next, 9 mg, 18 mg, 27 mg, 36 mg, and 45 mg MXenes were dissolved in 25 mL 1 M HClO_4_ solution (solution A), respectively. Afterwards, 136.7 μL aniline was dropped into solution A with a pipette gun to fully ultrasonically disperse it (solution B). Then, solution B was precooled in a freezer for 20 min. This was followed by 0.22801 g ammonium persulfate (APS) being weighed and added into a flat-bottomed centrifuge tube, with 5 mL 1 M HClO_4_ added to fully disperse it (solution C, which was also precooled in a freezer for 20 min). The HJ-4A magnetic stirrer was placed inside the freezer, and the temperature was adjusted from −3 to 3 °C. At the end of precooling, a magnet was added into solution B and fixed on a magnetic stirrer to start stirring. While solution B was stirred, solution C was quickly added to it for a low-temperature reaction for 20 h. At the end of the reaction, the reaction solution was washed with a large amount of methanol and 0.1 M HClO_4_ and freeze-dried to obtain MXene@PANI powder.

### 2.4. Preparation of MXene Ink and MXene@PANI Ink

The prepared MXene aerosol was dissolved in deionized water without any additives and ground for 2 h to obtain the MXene ink (MXene concentration of 400 mg/mL). The prepared MXene@PANI powder was dissolved in deionized water without any additives and ground for 2 h to obtain the printable MXene@PANI ink (MXene@PANI concentration of 300 mg/mL).

### 2.5. Fabrication of PVA/Na_2_SO_4_ Gel Electrolyte

PVA (9.09 g) was added into 100 mL deionized water in a beaker to obtain 10 wt.%. In order to prevent the evaporation of deionized water, the beaker was sealed with tin foil. Then, the beaker was moved to a water bath and stirred at 95 °C for 2 h using a water bath pot and a mechanical stirrer (LC-OES-60SH from Shanghai Licheng Bangxi Instrument Co., Ltd., Shanghai, China). While stirring, 20 mL 1M Na_2_SO_4_ solution was added. After being stirred evenly, the solution was then stood still for 8 h, and the upper bubbles were removed to obtain the PVA/Na_2_SO_4_ gel electrolyte.

### 2.6. Fabrication of MSCs

MXene ink and MXene@PANI ink were printed into three-dimensional thick interdigital electrodes on polyethylene terephthalate film (PET) substrates by a flexible electronic printer (mp1100 from Shanghai Mifang Electronic Technology Co., Ltd., Shanghai, China). In order to facilitate electrochemical testing, after printing one layer, we placed two pieces of nickel tape at the electrode feet and printed the ink layer by layer to fix it. The PVA/Na_2_SO_4_ gel electrolyte was then drop-cast onto the interdigital electrodes to fabricate MXene MSCs and MXene@PANI MSCs. In order to prevent the MSCs from any contact with air and affecting the electrochemical performance, we encapsulated MSCs with aluminum–plastic films.

### 2.7. Material Characterization

The surface morphologies of the as-prepared samples were inspected by a high-resolution field-emission scanning electron microscope (FESEM, SU8020, Hitachi High-Tech Corporation, Tokyo, Japan), while elemental mappings were obtained by energy-dispersive X-ray spectrometry (EDS). The crystal structures of the as-prepared samples were characterized by X-ray diffraction (XRD) analysis using an X-ray diffractometer (Rigaku Ultima IV from BRUKER AXS GMBH, Berlin, Germany). The composition of samples was further analyzed by X-ray photoelectron spectroscopy (XPS, Kratos Company, AXIS Ultra, San Diego, CA, USA). All the binding energies were referenced to the C 1 s line at 284.8 eV from adventitious carbon. Raman spectroscopy was performed on LabRAM (HORIBA HR800), and the excitation wavelength of the laser source was 532 nm. The rheological properties of the MXene@PANI ink were tested at room temperature with a rotary rheometer (TA Instruments-Waters LLC, HR 20, New Castle, DE, USA). The function of the apparent viscosity and the shear rate were recorded by the dynamic frequency scanning mode. The storage modulus (G′) and loss modulus (G″) were measured by the dynamic stress scanning mode at a fixed frequency of 1 Hz.

### 2.8. Electrochemical Measurements

The fabricated MSCs were measured in an electrochemical workstation (Chenhua CH760E, Shanghai, China) and cell testing system (LANHE, Wuhan, China) using a two-electrode system. Cyclic voltammetry (CV), electrochemical impedance spectroscopy (EIS), galvanostatic charge–discharge (GCD) curves, and cycling stability were obtained at room temperature.

The volumetric capacitances were used to evaluate the electrochemical performance of the printed MSCs. Cell capacitance (C) was derived from the GCD profiles, according to the following equations.

(1) $$C=\frac{I\times \Delta t}{\Delta V}$$

where I is the discharge current (A), Δt is the discharge time (s), and ΔV is the discharge potential (V).

The specific volumetric capacitance (*C_vol_*, F/cm^3^) of the MSCs was obtained by the following Equation (2):

(2) $$C_{vol}=\frac{C}{V}$$

where V is the total geometric volume of the MSCs, involving the positive and negative electrodes and the electrodes’ interspace, respectively.

The charge-storage kinetic analysis was carried out from the CV curves using the following equation:

(3) $$I=av^{b}$$

where *a* and *b* are determined from the slope of the peak current (i) versus the scan rate (v).

The diffusion control and potential of the dependent capacitive contributions were calculated using the relationship as follows:

(4) $$i=k_{1}v+k_{2}v^{1/2}$$

where $k_{1}$ and $k_{2}$ are constants representing the surface-controlled and diffusion-controlled capacity coefficients, respectively.

The volumetric energy density (*E*, Wh/cm^3^) and power density (*P*, W/cm^3^) of the MSCs were calculated based on Equations (3) and (4) as follows:

(5) $$E=\frac{0.5\;\times \;C_{\mathrm{vol}}\;\times \;{\Delta V}^{2}}{3600}$$

(6) $$P=\frac{3600\;\times \;E}{\Delta t}$$

## 3. Results and Discussion

### 3.1. Fabrication and Characterization of MXene@PANI Composites

**Figure 1:**
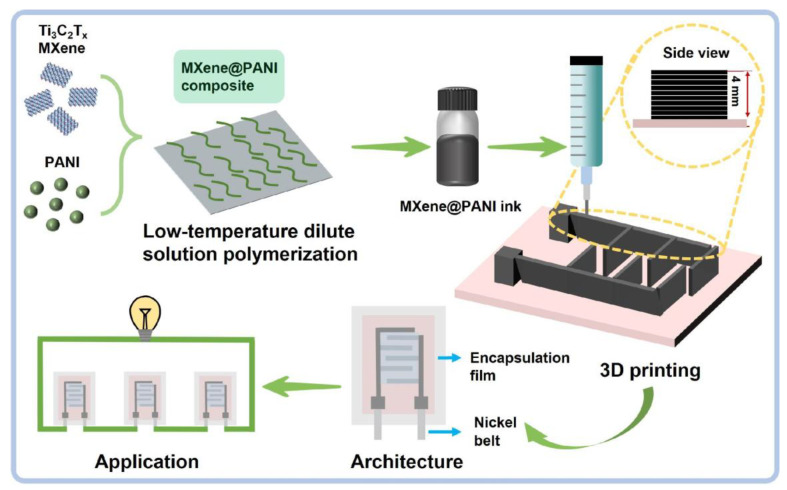
Fabrication schematic of the MXene nanosheet and the MXene@PANI nanofiber composite, additive-free composite ink, integrated electrode structures, MXene@PANI MSCs, and their potential applications.

To realize MXene@PANI electrodes with a 3D architecture, 2D Ti_3_C_2_T_x_ MXene nanosheets with single-layered structures were first prepared by a classical minimally intensive layer delamination (MILD) method [32,33], which brings about a large number of surface-active sites. To impede these nanosheets restacking due to van der Waals forces and further increase specific surface areas, PANI nanofibers were grown on the MXene nanosheets by low-temperature dilute solution oxidative polymerization, as shown in Figure 1. The hybridization of MXene nanosheets and pseudo-capacitance PANI is anticipated to enable the simultaneous contribution of electrical double-layer capacitance (EDLC) and pseudo-capacitance to final capacitance. In this regard, the formed MXene@PANI composites with a delicate assembly of 1D and 2D nanostructures were prepared in additive-free ink and then printed into the 3D integrated electrode structures on the flexible substrate (Figure 1), whose thickness could be tailored through the regulation of printing parameters. Accordingly, artificially designed 3D electrode structures with hierarchical structures were achieved, enabling the construction of the high mass loading of the active substance. These electrodes could be encapsulated with sealing films to form a larger variety of SCs with different sizes for the energy supply of electronic devices.

**Figure 2:**
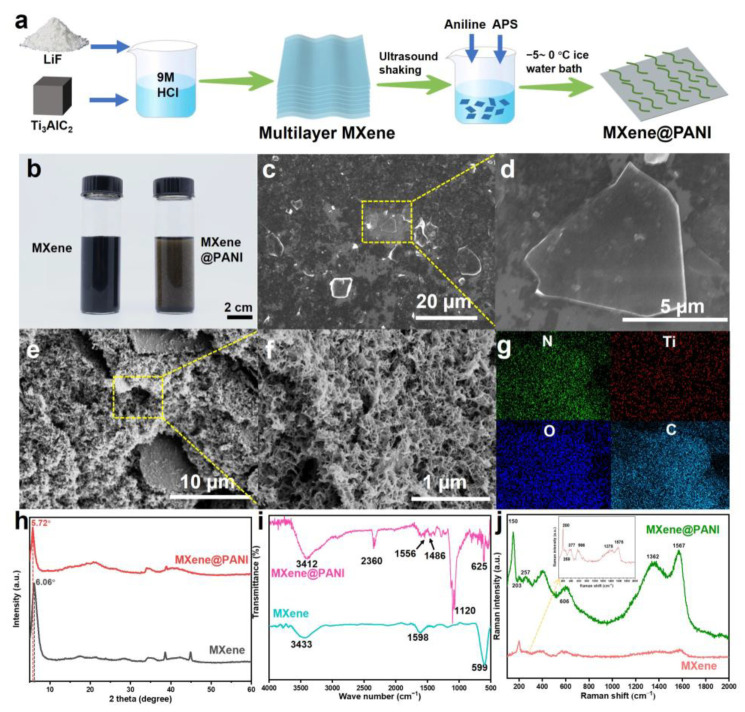
Structures and composition of the prepared MXenes and MXene@PANI composites. (**a**) Preparation illustration of single-layer MXene dispersion and MXene@PANI composites. (**b**) Optical photographs of the aqueous solution of MXenes and MXene@PANI composites. SEM images of (**c**,**d**) single-layer Ti_3_C_2_ MXene nanosheets and (**e**,**f**) MXene@PANI composite nanosheets at low and high magnification. (**g**) EDS mapping images of the N, C, O, Ti elements of MXene@PANI composites. (**h**) XRD patterns, (**i**) FTIR spectra, and (**j**) Raman spectra of single-layer Ti_3_C_2_ MXenes and MXene@PANI composites.

Considering the importance of the 2D MXene@1D PANI structure, the preparation detail of the MXene@PANI composites is given in Figure 2a. The single-layer Ti_3_C_2_T_x_ MXene aqueous solution was obtained through the etching treatment of the MAX phase with LiF and HCl, followed by ultrasonic delamination. The resultant single-layer MXene is dark green in color (Figure 2b left) and exhibits a clear Tyndall effect (Figure S1a). After freeze-drying, the aerogel MXene was obtained (Figure S1b). Subsequently, MXene@PANI composites were prepared by low-temperature dilute solution oxidative polymerization. Specifically, the different contents of the MXene aerosol were dispersed in 1 M HClO_4_ solution under low-temperature stirring (−3 to 3 °C). After the addition of aniline and precooled ammonium persulfate (APS) solution to react for 2 h, PANI nanofibers gradually grew on the MXene nanosheets to form the MXene@PANI composites with a reaction time beyond 9 h. This is verified by the fact that the dark green solution turns into a bright green color, as shown in Figure 2b, similarly to the successful growth of the PANI nanofibers on the arbitrary substrates previously [34]. To evaluate the successful preparation of 2D MXene@1D PANI composites, the morphology and structure of pure Mxene nanosheets and MXene@PANI composites were compared through scanning electron microscopy (SEM), as illustrated in Figure 2c–f. As can be seen in the SEM images of MXenes (Figure 2c,d), the MXenes exhibit a smooth surface without obvious oxides, suggesting no oxidation. When growing the PANI nanofibers, it can be observed that the 2D MXene nanosheets are all covered by 1D PANI nanofibers to form 3D structures (Figure 2e,f and Figure S2a–c), despite MXene contents ranging from 9 mg to 45 mg. However, the MXene content of 36 mg enabled the neatly arranged and moderate 1D PANI nanofibers to grow on the 2D MXene nanosheets, as compared with the highly random structure at other MXene contents (Figure S2a–c). This might be ascribed to the fact that when the MXene content is less than 36 mg, aniline not only polymerizes on the surface of the MXene nanosheets but also self-nucleates into randomly arranged PANI nanofibers in the bulk solution. When the MXene content is more than 36 mg, the PANI nanofibers cannot completely cover the MXene nanosheets. However, when the MXene content is 36 mg, these PANI nanofiber arrays are uniformly distributed on MXene nanosheets, as supported by homogeneous distributions of Ti, C, O, and N elements on the MXene surface from the EDS mapping images in Figure 2g. This indicates that nucleation and growth only occurred on the surface of the MXene nanosheets [35]. In order to further elucidate the surface characteristics of the three-dimensional composite structure composed of the MXene nanosheets and PANI nanofibers, we conducted AFM tests on the MXene nanosheets and MXene@PANI composites. As shown in Figure S3, the surface of the MXene nanosheets is densely covered with regularly aligned PANI nanofibers. Furthermore, the thickness of the MXene nanosheets increases from approximately 3.5 nm to about 40 nm after the PANI nanofibers are grown on their surface. Therefore, MXene@PANI composites were successfully achieved, among which the construction of a 1D structure greatly improves specific surface area of MXenes.

To further demonstrate the rational hybridization of different components, the composition of the obtained samples was characterized by X-ray diffraction (XRD), Fourier transform infrared (FTIR), Raman, X-ray photoelectron spectroscopy (XPS), and Brunauer–Emmett–Teller (BET). As given in Figure 2h and Figure S4a, the MXene nanosheets and MXene@PANI composites have the same diffraction peak as that of Ti_3_AlC_2_ MAX, despite it moving from 9.5° to 5.72°. Moreover, the diffraction peak of the pristine PANI at around 25° indexing to the amorphous phase (Figure S4b) has also been imparted to the MXene@PANI composites. These results indicate the successful complexation of MXenes and PANI, which has been reported in previous publications [36,37]. In addition, as PANI grows on MXene nanosheets, the (002) peak shifts to small diffraction angles, indicating large facet spacings after the growth of PANI nanofibers on MXene nanosheets [38]. Figure S5 shows N_2_ adsorption–desorption isotherms. As can be seen, the pure MXenes and MXene@PANI composites exhibited a type of IV isotherm with H3-type hysteresis loops in the range of 0.1–1.0 P/P_0_, manifesting the existence of a mesoporous structure. Additionally, the hysteresis loops sharply increased absorption at P/P_0_ close to one, suggesting the mesoporous–macroporous hybrid structure (the inset in Figure S5). If the MXene nanosheets are restacked, they will produce slit-shaped pores, which are different from the shapes of the pores of the MXenes and MXene@PANI composites we prepared, suggesting the prepared MXenes and MXene@PANI composites are not restacked. The calculated BET surface area of the pure MXenes was 6.8 m^2^/g, while increasing to 7.5 m^2^/g after compositing with PANI. Such a slight increase in the surface area is attributed to the growth of 1D PANI nanofibers on the surface of 2D MXene nanosheets. The BET measurements were conducted prior to the ink formulation process. Therefore, densification during ink preparation did not affect the results. Furthermore, multiple BET measurements were performed, and the obtained specific surface area values showed good consistency, effectively eliminating potential measurement errors. As MXenes are a 2D material with an intrinsic specific surface area, the growth of nanofibers on their surface contributes minimally to the overall surface area. However, these nanofibers play a crucial role in preventing the restacking of MXene nanosheets. Additionally, the synergy between PANI and MXenes contributes to a comprehensive and immediate electrochemical reaction at the electrolyte–electrode interface, leading to a substantial increase in the capacitance of MXene@PANI micro-supercapacitors. Consequently, the final composite material exhibited significantly enhanced capacity compared to that of the polymer-free counterpart. Additionally, these PANI nanofibers are firmly grown on the MXene surface due to the formation of weak interaction between the aniline monomers and functional groups of the MXene nanosheets, as supported by the peak position shifting of the MXene@PANI composites compared with that of MXenes in the Fourier transform infrared (FTIR) spectra (Figure 2i), which also verified the successful introduction of PANI nanofibers. To further evaluate component hybridization, Raman spectroscopy was employed to analyze the prepared samples (Figure 2j). For MXenes, the strong peaks are situated around 200 cm^−1^, 259 cm^−1^, 377 cm^−1^, and 566 cm^−1^, which correspond to the vibration of Ti-C [39]. For MXene@PANI, the peaks at 150 cm^−1^, 203 cm^−1^, 257 cm^−1^, and 606 cm^−1^ can be indexed to the following vibrational modes: E_g_ of bare Ti_3_C_2_, A_1g_ of Ti_3_C_2_O_2_, E_g_ of Ti_3_C_2_F_2_, and E_g_ of Ti_3_C_2_(OH)_2_, respectively. The results demonstrated the presence of -O, -OH, and -F active groups on the surface of pristine Ti_3_C_2_T_x_ MXenes. Furthermore, distinctive peaks are found for the D and G bands in the MXenes and MXene@PANI composites, where the intensity of the D band is comparable to that of the G band. The calculated ratios of I(D) to I(G) for the MXenes and MXene@PANI composites are about 0.95 and 0.91, suggesting that the MXenes and MXene@PANI composites have ordered crystal structures and a certain number of defects [40]. The results of XPS are shown in Figure S6, and the survey clearly shows the presence of F, O, Ti, and C elements on the MXenes and MXene@PANI composite, while N elements are newly present on the MXene@PANI composite. In the high-resolution spectra of the Ti, C, O, and F elements, it can be found that the peaks of the MXene@PANI composite exhibit a slight shift to a higher binding energy direction compared with those of the pure MXenes (Figure S6b–e), which may result from the chemical interaction between the PANI nanofibers and the Ti_3_C_2_T_x_ nanosheets. The high-resolution N1s spectrum of the MXene@PANI composite (Figure S6f) can be decomposed into four predominant peaks corresponding to four kinds of nitrogen bonds of PANI: Ph-N=Ph at 398.4 eV, Ph-NH-Cl at 400.1 eV, Ph-NH-Ph at 401.2 eV, and Ph-N-F at 402.4 eV, verifying the existence of PANI in MXene@PANI [41,42], demonstrating the successful combination of PANI and MXenes. The above results demonstrate that aniline has been successfully polymerized into PANI on the surface of pristine Ti_3_C_2_T_x_ MXenes. Such a polymerization not only protects MXene nanosheets from oxidation and re-accumulation but also increases active sites and triggers a synergistic effect of the component and structure, finally enhancing its electrochemical properties.

### 3.2. Preparation and Regulation of MXene@PANI Composite Ink

**Figure 3:**
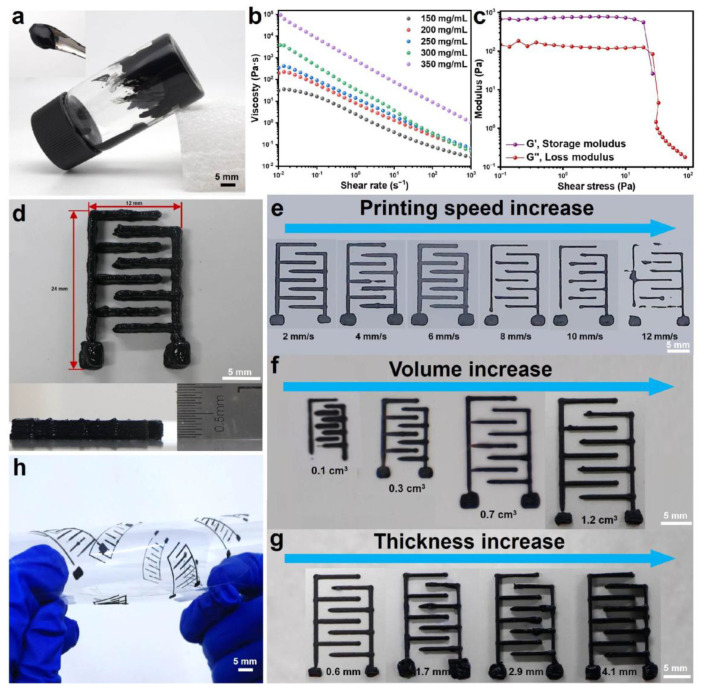
Printability of the MXene@PANI composites. (**a**) Optical photograph of the MXene@PANI composite ink. (**b**) Apparent viscosity as a function of the shear rate and (**c**) G′ and G″ as a function of the shear stress for the MXene@PANI ink. (**d**) Interdigital electrode with a thickness of 4.1 mm printed with the MXene@PANI composite ink. (**e**) Regulation of the print speed. (**f**) Different precision and (**g**) thickness printed with the MXene@PANI composite inks. (**h**) Printed electrodes on flexible substates.

Given the limitation of traditional coating electrodes and the merits of 3D printed microelectrodes, the MXene@PANI composites needed to be formed into water-based inks with excellent self-supporting and shear-thinning properties for the electrodes with high mass loading (Figure 3a). For this purpose, five composite inks, with different concentrations of 150 mg/mL, 200 mg/mL, 250 mg/mL, 300 mg/mL, and 350 mg/mL were prepared and regulated without the additives. The viscoelastic properties of the MXene@PANI composite inks were characterized through viscosity and oscillation testing. It was observed that all ink viscosities change with the shear rate (Figure 3b). When the MXene@PANI composite ink concentrations are 300 mg/mL and 350 mg/mL, the viscosities reach up to 3745.34 Pa·s and 93,874.6 Pa·s, suggesting excellent printability and self-supporting properties. Moreover, the shear-thinning behavior of the composite inks with a concentration of 300 mg/mL and 350 mg/mL is evident, as depicted in Figure 3c and Figure S7 showing the storage modulus (G′) and loss modulus (G″) as functions of shear stress. The plateaus of G′ for 300 mg/mL and 350 mg/mL are close to the value of 10^3^ and 10^5^, which are approximately one order of magnitude higher than those of G″ whose plateau are close to the value of 10^2^ and 10^4^. It can be inferred that the inks with concentrations of 300 mg/mL and 350 mg/mL behave as an elastic solid, which is better than those with concentrations of 150 mg/mL, 200 mg/mL and 250 mg/mL, as further demonstrated by the printed patterns (Figure S8). However, the yield stress (i.e., the stress at G′ = G″) of the 350 mg/mL ink is too high (720.083 Pa) to be extruded from the syringe. Consequently, the ink with a concentration of 300 mg/mL was anticipated to possess excellent printability and superior self-supporting properties, which was selected as the concentration of the MXene@PANI composite ink for subsequent electrode printing.

### 3.3. Regulation of Printing Parameters

To obtain the desired 3D electrodes with different structures, printing parameters (such as the printing speed as well as the size and thickness of the electrode) were also considered when the ink was fixed. Therefore, the printing speed, size, and layer number were regulated to achieve 3D thick microelectrodes with a tiny volume (Figure 3d). Usually, a high printing speed is conducive to an improvement in production efficiency, while a speed that is too high is not conducive to continuous printing. To this end, different printing speeds ranging from 2 mm/s to 12 mm/s were employed firstly to prepare the same pattern. As observed in Figure 3e, all the printed samples showed almost clear patterns on the whole. However, the printed patterns became complete with even lines when the printing speed increased to 6 mm/s and then became incomplete as the speed increased. When the printing speed was 6 mm/s, the printed integrated pattern showcased the most uniform ink, smoothest line, and highest integrity (Figure 3e). This might be attributed to the fact that a proper speed results in the uniform filamentary morphology of the selected ink extruded through the needle. To explore the printing minimum limitation, the patterns with different sizes were prepared based on the above printing parameter and the selected inks, as shown in Figure 3f. The resolution of the pattern deteriorated when the printing area reached 0.1 cm^3^. As the printing size increased, the resolution of the printed pattern became higher, and the electrode structure was presented more completely. With a further increase, the lines of the electrode pattern got thinner and the precision became decreased, which was unsuitable for the preparation of microelectrodes. In view of the electrode requirement for thickness, the patterns with a different thicknesses need to be evaluated in conjunction with the electrochemical performance below. As given in Figure 3g, with the printed thickness increasing, the printed structures and patterns presented a slight deformation, since the increase in thickness caused tiny collapses of the electrode structure. When printing at a thickness of 0.6 mm, the printing effect is the best, but the selection of the specific thickness needs to be considered in combination with the electrochemical performance of the electrodes. When the printing speed is fixed at 6 mm/s, various patterns (including GXU, Chinese characters of Guangxi University, concentric circles, puppies, and snowflakes) can be printed smoothly (Figure S9a–d), indicating the ink we prepared has good printability. By measuring the thickness of the three parts of the MXene@PANI electrode, we obtained three thickness values of 4.2 mm, 4.1 mm, and 4.1 mm, respectively, with an average thickness of 4.13 mm and a square difference of 0.05774 mm, indicating that the thickness of the electrode we printed was homogeneous (Figure S9e). These things considered, the composite inks were printed on PET flexible substrates. Even when bending at various angles, no cracks appeared on the substrate, and the ink did not fall off (Figure 3h), showing excellent flexibility and holding promising potential for flexible electronics.

Based on the regulation of printing structure quality, the effect of printing parameters on the electrochemical performance of printed electrodes was further determined, including the printed thickness and volume of electrodes. To determine the proper potential window for electrochemical evaluation, different potential windows were utilized to perform cyclic voltammetry (CV) testing. With the voltage increasing from 0.6 V to 1.2 V, the integral area of the CV curve ascends without obvious polarization (Figure S10). However, voltages above 1.23 V might result in hydrolysis of the hydrogel electrolyte. Thus, the potential window of 0–1.2 V was selected for further electrochemical characterization. In theory, the increase in electrode thickness leads to an improvement in mass loading, thereby enhancing the electrochemical performance. As demonstrated, the integrated area of the CV curve of electrodes with different thicknesses (0.6 mm, 1.7 mm, 2.9 mm, and 4.1 mm) increases as the thickness of the electrode rises (Figure S11). The maximum volumetric capacitance was obtained when the electrode thickness was 4.1 mm, which indicates that the thickness is greatly dominated by the printed structure quality. If better quality of electrode structures is achieved, the higher capacitance is obtained. Therefore, we chose 4.1 mm as the thickness for electrode printing. Except for electrode thickness, other electrode sizes were regulated. Keeping at this thickness, with the increase in electrode volumes from 0.3 cm^3^ to 1.2 cm^3^, the volumetric capacitance gradually rises and then keeps at about 360 mF/cm^3^ (Figure S12). This indicates that the electrode with a smaller size is more conducive to the realization of higher capacitance, holding huge potential for miniaturized energy supply. Therefore, we chose 0.7 cm^3^ as the volume for electrode printing.

### 3.4. Assembly and Electrochemical Performance Testing of MMSCs and MPMSCs

**Figure 4:**
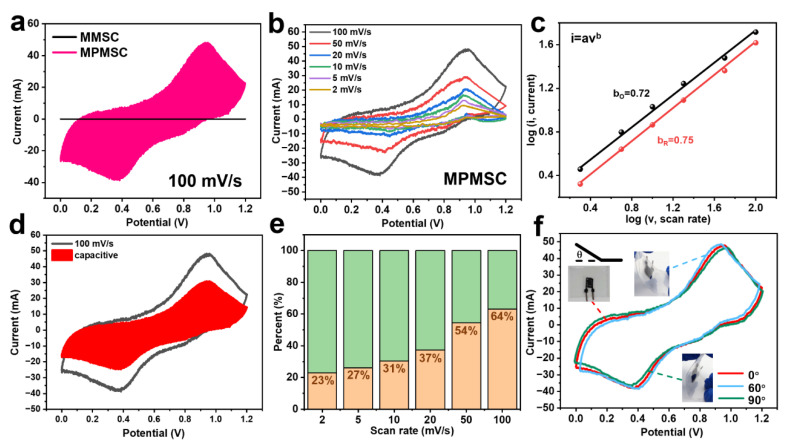
CV curves and capacitance contribution of MSCs based on the MXenes and MXene@PANI composites. (**a**) CV curves of the MMSCs and MPMSCs at 100 mV/s. (**b**) CV curves of the MPMSCs at different scanning rates. (**c**) Logarithm plot of the peak currents of the MPMSCs as function of the scan rates. (**d**) CV profile at 100 mV/s with the red area representing the surface capacitive contribution. (**e**) Normalized capacity contribution at various scan rates. (**f**) CV curves of the MXene@PANI composite MSCs at a scan rate of 100 mV/s at different bending angles.

Utilizing the above regulated parameters, MSCs with pure MXenes and MXene@PANI composites, denoted as MMSCs and MPMSCs, were fabricated through the introduction of gel electrolytes and sealing films, whose performance was explored systematically. As given in Figure 4a, the CV curves of the MMSCs and MPMSCs display a significant difference at a scanning rate of 100 mV/s. The MMSCs exhibit a nearly rectangular CV profile, implying the electrical double-layer capacitance of MXenes, whereas the MPMSCs display an obvious pseudo-capacitance CV curve with two pairs of redox peaks, which originates from the redox transitions of PANI. This pseudo-capacitance contribution makes the capacitance enhancement of the MSCs. With the scanning rate increasing from 2 mV/s to 100 mV/s, the CV curve of the MPMSCs illustrates an enlarged enclosed area while retaining a respective profile (Figure 4b). Nonetheless, the nearly rectangular CV curve of the MMSCs is distorted with larger polarization, which should be ascribed to the establishment of an electrical double-layer without good matching with the fast scanning rate (Figure S13). Therefore, the synergistic capacitance contribution from the Faradaic reactions and the electrical double-layer of the MXene@PANI composite is far superior to that of pure MXenes, signifying the performance improvement. To determine these contributions, the charge-storage kinetic analysis of the MPMSCs was carried out based on the equation $I=av^{b}$, where “a” and “b” are determined from the slope of the peak current (*I*) versus the scan rate (*v*). The “b” value of 0.5 is indicative of a diffusion-controlled process, while the “b” value of 1.0 signifies a surface capacitive case [19,43,44]. As shown in Figure 4c, the “b” value corresponding to two peak currents was calculated as 0.72 and 0.75, respectively, which are between 0.5 and 1, suggesting a hybrid energy storage mechanism originated from the concurrent contribution of both pseudo-capacitance and surface capacitance.

To quantify the hybrid energy storage mechanism of the MPMSCs, a capacitive contribution analysis was conducted across the total current. The CV curves were fitted with the equation as follows: $i=k_{1}v+k_{2}v^{1/2}$, where $k_{1}$ and $k_{2}$ are constants representing the surface-controlled and diffusion-controlled capacity coefficients, respectively. The fitting of contributions was carried out over a scan rate range of 2–100 mV/s, and the resulting contribution ratios are presented in Figure 4d,e. As the scan rate increases, the surface-capacitive contribution becomes more dominant in the energy storage of the MPMSCs. The surface-capacitive contribution increased from 23% to 64% when the scan rate increased from 2 mV/s to 100 mV/s. This means that the kinetics and charge storage at the core of the capacitance in the MPMSCs are relatively fast at high scanning rates [20]. To prove the durability of the MPMSCs for flexible energy storage, the electrochemical performance was evaluated under different bending conditions at 100 mV/s. As given in Figure 4f, the CV curves almost show no changing profiles under the bending of 60° and 90°. This indicates that the MPMSCs show exceptional electrochemical stability regardless of the degree of bending, suggesting good flexibility.

**Figure 5:**
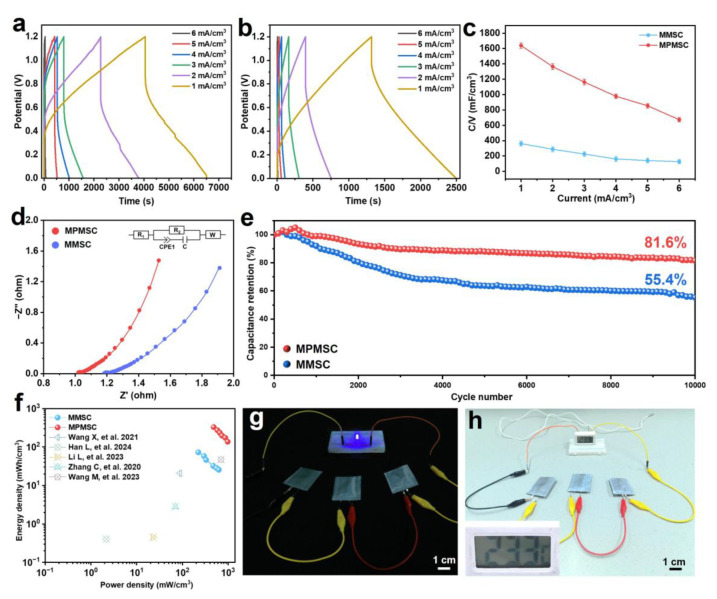
Electrochemical performance of MSCs based on the MXenes and MXene@PANI composites. GCD profiles of the (**a**) MPMSCs and (**b**) MMSCs. (**c**) Volumetric capacitance of the prepared MSCs at various current densities. (**d**) Nyquist plots of the prepared MSCs. (**e**) Cycling stability of the prepared MSCs at 2 mA/cm^3^ over 10,000 charging/discharging cycles. (**f**) Ragone plots of MSCs in comparison with other reported devices in terms of energy and power densities [21,22,32,46,47]. (**g**,**h**) Optical images of the MPMSCs lighting up a bulb and powering up electronic devices.

To quantify the capacitance accurately, the superior pseudocapacitive behavior of the prepared MSCs was determined by the GCD profiles. As illustrated in Figure 5a,b, the discharge time of the MSCs shortens with the current density increasing. Due to the synergistic contribution of the electrical double-layer capacitance of MXenes and the pseudo-capacitance of PANI, the MPMSCs show much longer charging and discharging times than those of the MMSCs, thereby generating larger capacitance. As calculated in Figure 5c, both volumetric capacitances change with the current density, while the capacitance of the MPMSCs is significantly higher than that of the MMSCs. At the low current density of 1 mA/cm^3^, the capacitance of the MPMSCs is 1638.3 mF/cm^3^, about 4.5 times that of the MMSCs (361.9 mF/cm^3^). Even at 6 mA/cm^3^, a high volumetric capacitance of up to 672.9 mF/cm^3^ is still retained, which is four times higher than that of MMSCs. Such excellent capacitance can be attributed to how the combination of MXenes and PANI contributes cooperatively to an improvement in capacitance. The MXene@PANI composite features a 3D architecture constructed by the growth of 1D PANI nanofibers on the surface of 2D MXene nanosheets. This hierarchical structure design not only shortens the ion transport pathways but also enhances the stability of the electrode material. The conjugated structure of PANI improves electron delocalization, resulting in superior conductivity and high capacitance. Meanwhile, the pseudo-capacitance feature of PANI enables it to contribute a high Faraday pseudo-capacitance for the electrode. Furthermore, the 3D structures constructed by 1D PANI nanofibers and 2D MXene nanosheets can promote the transport of electrolyte ions. Eventually, the combination of MXenes and PANI significantly improves the electrochemical performance of the electrode material [21]. To verify the conductivity improvement, the ion diffusion and charge-transfer properties of the prepared MSCs were evaluated using electrochemical impedance spectroscopy (EIS) tests. As seen in the EIS curve (Figure 5d), it consists of a tiny semicircle and a sloped line. The presence of semicircles indicates that the fitting equivalent circuit of MSCs does not contain a parallel resistance–capacitance, which is related to non-ideal capacitive behavior. Accordingly, a constant phase element (CPE) is selected [45]. An equivalent circuit diagram is given with the first R_1_ representing the solution resistance, C indexing the electrostatic double-layer capacitance, R_2_ denoting the charge-transfer resistance, and W indicating the diffusion-controlled impedance. The MPMSCs exhibit a larger slope and lower internal resistance than those of the MMSCs. This not only indicates the minimum charge-transfer resistance and optimal ion diffusion/transportation kinetics but also suggests an improved rate of mass transfer for ions during electrochemical processes [36]. These features are favorable for faster Faradaic reactions, leading to excellent capacitance.

Given the long-lasting operation in practical scenarios, the cycle stability is pivotal for the evaluation of MSCs. We have conducted a comprehensive stability assessment of the material after 10,000 charging/discharging cycles to explore its durability. As shown in repeated charging/discharging measurements at 2 mA/cm^3^ (Figure 5e), the capacitance retention rate of the MPMSCs is 81.6% after 10,000 charging/discharging cycles, overwhelming the MMSCs (only 55.4%). In addition, to investigate the impact of recycling on the structure of the MXene@PANI composite, we analyzed its surface morphology and crystal structure after use. The results of these analyses are illustrated in Figure S14. After 10,000 cycles of charging/discharging tests, the surface morphology of MXenes remained largely unchanged. The structure of the PANI nanofibers on the surface was intact, with no significant shedding observed (Figure S14a–d). This indicates the robustness of the combining method employed. Additionally, XRD analysis (Figure S14e) revealed that the peak shape of the composite material after 10,000 charging/discharging cycles is consistent with that of the fresh MXene@PANI material, and no oxidation peak of MXenes was detected. These findings further demonstrate the high efficiency and reliability of this composite structure. This good stability suggests that the capacitance is enhanced by the combination of 3D materials and the structure design. The energy and power properties, as key evaluating parameters for MSCs, are calculated from Equations (5) and (6) and given in the Ragone plot (Figure 5f). It shows that the MPMSCs deliver a maximum energy density of 328.2 mWh/cm^3^ at a power density of 482.2 mW/cm^3^ and a maximum power density of 972.8 mW/cm^3^ at an energy density of 137.1 mWh/cm^3^, which is obviously higher than previous reports (Table S1). To further demonstrate the performance of the MPMSCs, they were connected in series to supply a high output voltage to light a bulb (Figure 5g) and power a thermometer (Figure 5h). These results herald that our MPMSCs obtained by the 3D printing technique should be a highly promising candidate for high-performance power supply towards portable and wearable electronics.

## 4. Conclusions

In summary, a novel method was achieved successfully to prepare MXene@PANI composites with 1D PANI nanofibers on the surface of 2D MXene nanosheets to form a three-dimensional structure (with the temperature ranging from −3 °C to 3 °C), which offers abundant active sites and facilitates rapid ion transport. The MXene@PANI composites were subsequently synthesized into additive-free inks and then printed into MXene@PANI MSCs. The prepared MXene@PANI MSCs exhibited good flexibility and excellent electrochemical properties. The MXene@PANI MSCs show an excellent volumetric capacitance of 1638.3 mF/cm^3^ with a volumetric energy density of up to 328.2 mWh/cm^3^ at a volumetric power density of 482.2 mW/cm^3^ and a volumetric energy density of 137.1 mWh/cm^3^ at a volumetric power density of 972.8 mW/cm^3^. In addition, the MXene@PANI MSCs also possess good cycling stability, retaining 81.6% of the initial capacitance after 10,000 charging/discharging cycles at a current density of 2 mA/cm^3^, and its performance is far superior to that of the pure MXene MSCs, verifying a great improvement in electrochemical performance. This remarkable stability is attributed to the robust and well-designed hierarchical structure. The self-growth strategy allows PANI nanofibers to firmly anchor onto the surface of MXenes, thereby enhancing mechanical stability while simultaneously facilitating efficient charge transfer. Such MXene@PANI composite MSCs hold huge potential for powering miniatured electronics (such as wearable electronic devices, smart sensors), offering a promising solution for high-performance micro-supercapacitors.

## Data Availability

The original contributions presented in this study are included in the article/Supplementary Material. Further inquiries can be directed to the corresponding authors.

## Supplementary Materials

The following supporting information can be downloaded at: https://www.mdpi.com/article/10.3390/ma18102277/s1, Figure S1. (a) Tyndall effect of MXene aqueous solution. (b) Optical photograph of single-layer MXene; Figure S2. SEM images of MXene@PANI composites (the content of MXene was (a) 9 mg, (b) 18 mg, (c) 27 mg, and (d) 45 mg, respectively); Figure S3. AFM image and thickness distribution of single-layer MXene nanosheets and MXene@PANI composites; Figure S4. XRD patterns of (a) Ti3AlC2 MAX and (b) PANI; Figure S5. N2 adsorption/desorption isotherm and Pore volume of s-Ti3C2Tx MXene (green) and MXene@PANI composites (red); Figure S6. XPS spectrum of MXene and MXene@PANI composites: (a) XPS survey, (b) Ti 2p spectrum, (c) C 1s spectrum, (d) O 1s spectrum, (e) F 1s spectrum and (f) N 1s spectrum of MXene@PANI composites and MXene (MXene@PANI composites on top, MXene on bottom); Figure S7. Storage modulus (G’) and loss modulus (G”) as a function of shear stress for each MXene@PANI composite ink (the concentrations were (a) 150 mg/mL, (b) 200 mg/mL, (c) 250 mg/mL, (d) 300 mg/mL and (e) 350 mg/mL respectively); Figure S8. Optical photographs of interdigital electrodes printed with different concentrations of MXene@PANI composite ink; Figure S9. Printing of (a) English abbreviation and Chinese characters of Guangxi University, (b) concentric circles, (c) puppy and (d) snowflake patterns with MXene@PANI composite inks. (e) Measurement of MXene@PANI electrode thickness; Figure S10. Optimization of the voltage window. (a–d) CV curves in the 0–0.6 V, 0–0.8 V, 0–1.0 V and 0–1.2 V voltage window. (e) Comparison of CV curves for different voltage windows with a scan rate of 100 mV/s; Figure S11. Optimization of the thickness of electrode. (a,b) CV and GCD curves of 0.6 mm MXene electrode. (c,d) CV and GCD curves of 1.7 mm MXene electrodes. (e,f) CV and GCD curves of 2.9 mm MXene electrode. (g,h) CV and GCD curves of 4.1 mm MXene electrode. (i) The volumetric capacitance of MXene MSC of various thickness at different current densities; Figure S12. Optimization of electrode sizes. (a,b) CV and GCD curves of 0.3 cm^3^ MXene electrodes. (c,d) CV and GCD curves of 0.7 cm^3^ MXene electrode. (e,f) CV and GCD curves of 1.2 cm^3^ MXene electrode. (g) The volumetric capacitance of MXene MSC of various layers at different current densities; Figure S13. CV curves of MMSC at different scanning rates; Figure S14. SEM images of (a,b) initial MXene@PANI electrode and (c,d) MXene@PANI electrode after 10,000 charging/discharging cycles. (e) XRD pattern of initial MXene@PANI electrode and MXene@PANI electrode after 10,000 charging/discharging cycles; Table S1. Energy density and power density of different devices based on MXene/conductive polymers.

## Abbreviations

The following abbreviations are used in this manuscript:

1D	One-dimensional
2D	Two-dimensional
3D	Three-dimensional
MSCs	Micro-supercapacitors
PANI	Polyaniline
MBs	Micro-batteries
APS	Ammonium persulfate
m-Ti_3_C_2_	Multilayer Ti_3_C_2_
s-Ti_3_C_2_	Single-layer Ti_3_C_2_
PET	Polyethylene terephthalate
MILD	Minimally intensive layer delamination
EDLC	Electrical double-layer capacitance
SEM	Scanning electron microscopy
AFM	Atomic force microscopy
XRD	X-ray diffraction
FTIR	Fourier transform infrared
XPS	X-ray photoelectron spectroscopy
BET	Brunauer–Emmett–Teller
CV	Cyclic voltammetry
GCD	Galvanostatic charge/discharge
EIS	Electrochemical impedance spectroscopy
CPE	Constant phase element
MMSCs	MXene MSCs
MPMSCs	MXene@PANI MSCs
