# Supplementary material for: Three-Dimensional Printed MXene@PANI Hierarchical Architecture for High-Performance Micro-Supercapacitors
Source: Materials (Basel). 2025 May 14;18(10):2277. doi: 10.3390/ma18102277 (PMC12113580; doi:10.3390/ma18102277)
Supplement: Supplementary file 1 [file materials-18-02277-s001.zip › materials-3639928-supplementary.pdf]

materials

Supplementary Materials

Three-Dimensional Printed MXene@PANI Hierarchical Architecture for High-Performance Micro-Supercapacitors

Anyi Zhang [1], Yiming Wang [1], Haidong Yu [2,*] and Yabin Zhang [1,*]

[1] Guangxi Key Laboratory of Processing for Non-Ferrous Metals and Featured Materials, MOE Key Laboratory of New Processing Technology for Non-Ferrous Metals and Materials, School of Resources, Environment and Materials, Guangxi University, Nanning 530004, China
[2] School of Chemistry and Chemical Engineering, Guangxi University, Nanning 530004, China
* Correspondence: yuhaidong_chem@163.com (H.Y.); ybzhang@gxu.edu.cn (Y.Z.)

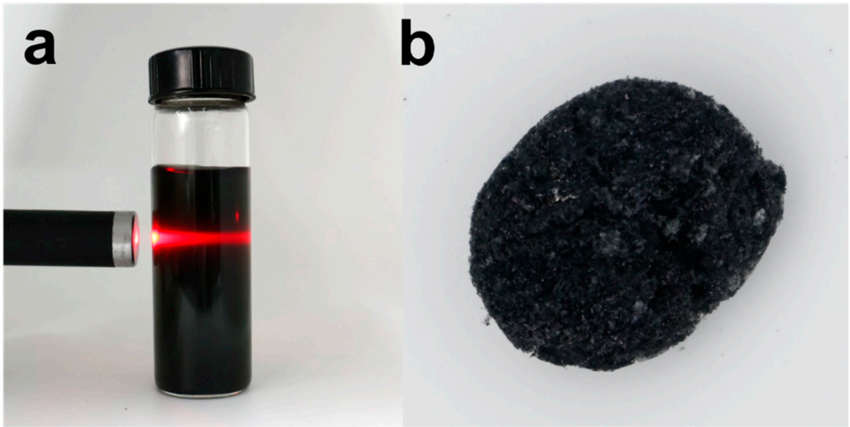

Figure S1. (**a**) Tyndall effect of MXene aqueous solution. (**b**) Optical photograph of single-layer MXene.

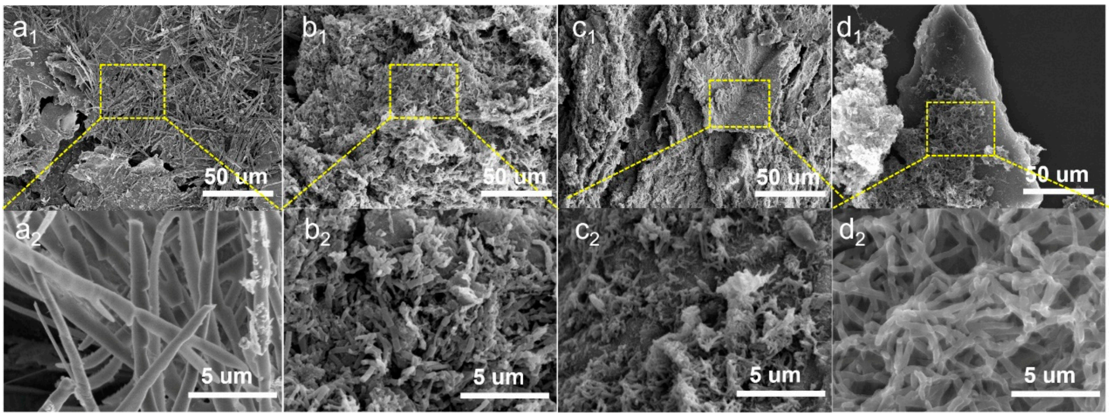

Figure S2. SEM images of MXene@PANI composites (the content of MXene was (**a**$_1$,**a**$_2$) 9 mg, (**b**$_1$,**b**$_2$) 18 mg, (**c**$_1$,**c**$_2$) 27 mg, and (**d**$_1$,**d**$_2$) 45 mg, respectively).

 https://doi.org/10.3390/ma18102277

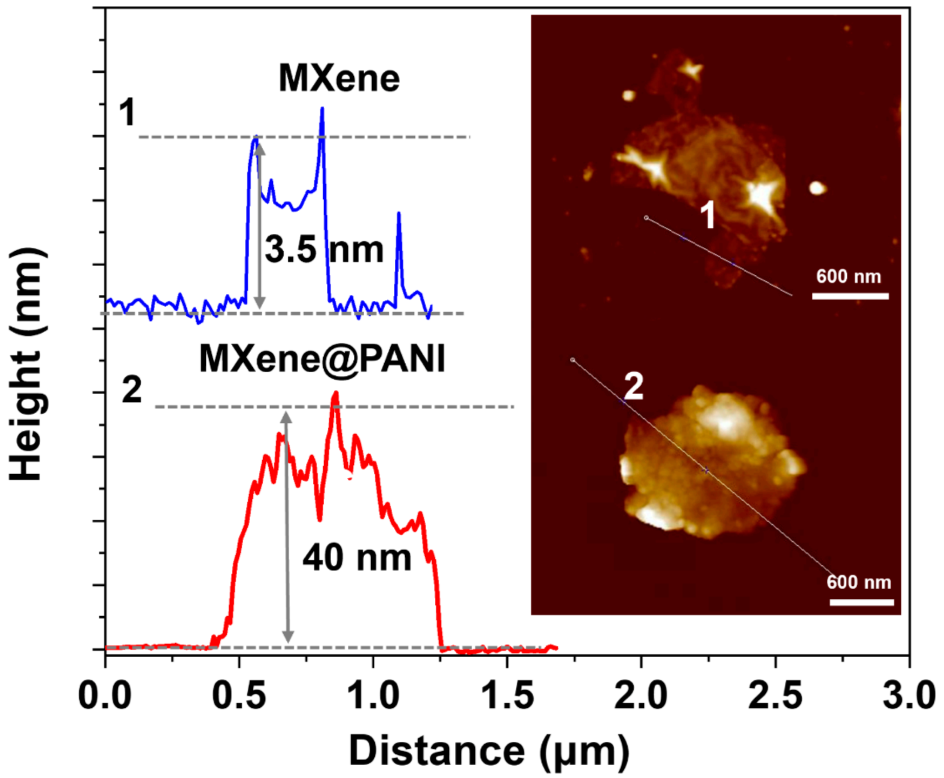

Figure S3. AFM image and thickness distribution of single-layer MXene nanosheets and MXene@PANI composites.

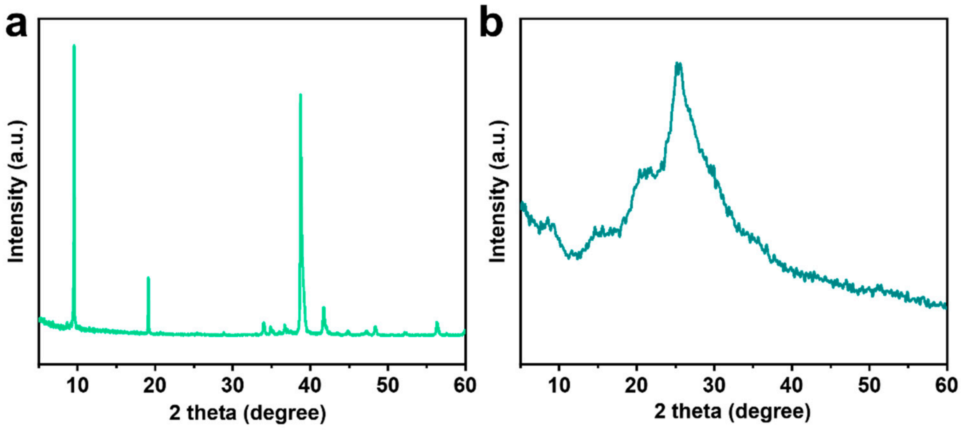

Figure S4. XRD patterns of (**a**) Ti_3AlC_2 MAX and (**b**) PANI.

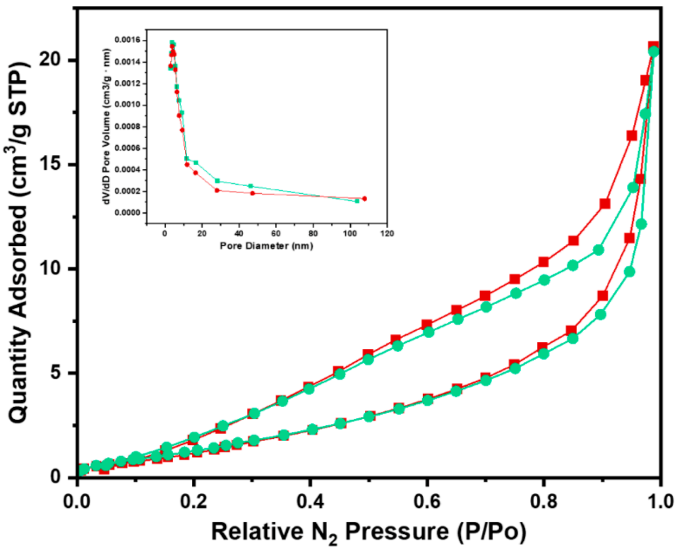

Figure S5. N_2 adsorption/desorption isotherm and Pore volume of s-$Ti_3C_2T_x$ MXene (green) and MXene@PANI composites (red).

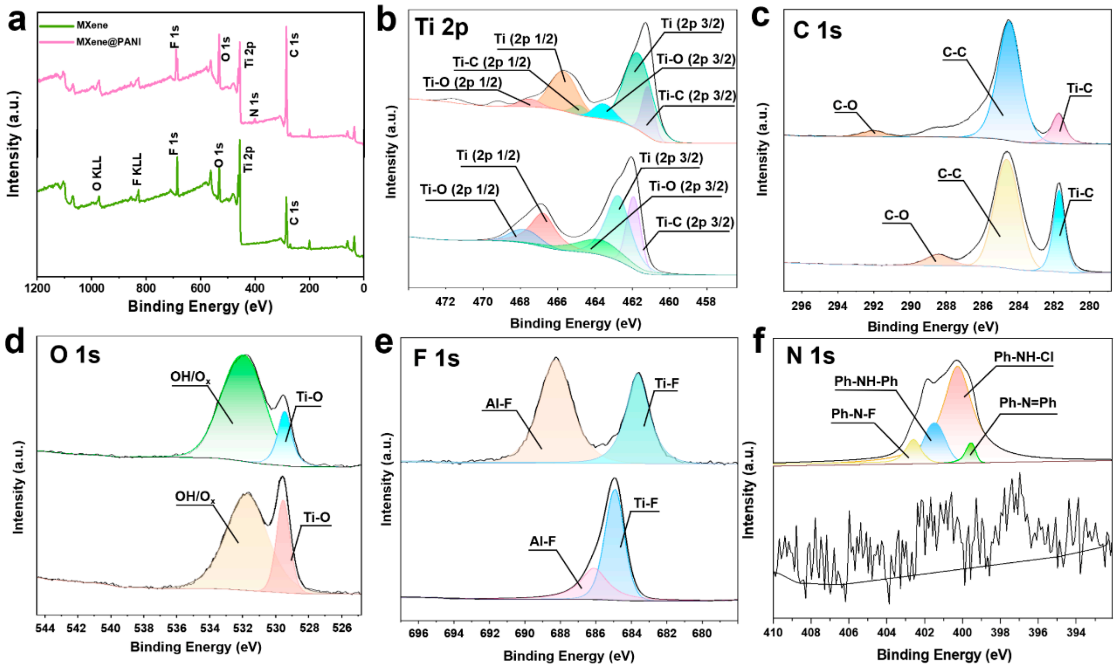

Figure S6. XPS spectrum of MXene and MXene@PANI composites: (**a**) XPS survey, (**b**) Ti 2p spectrum, (**c**) C 1s spectrum, (**d**) O 1s spectrum, (**e**) F 1s spectrum and (**f**) N 1s spectrum of MXene@PANI composites and MXene (MXene@PANI composites on top, MXene on bottom).

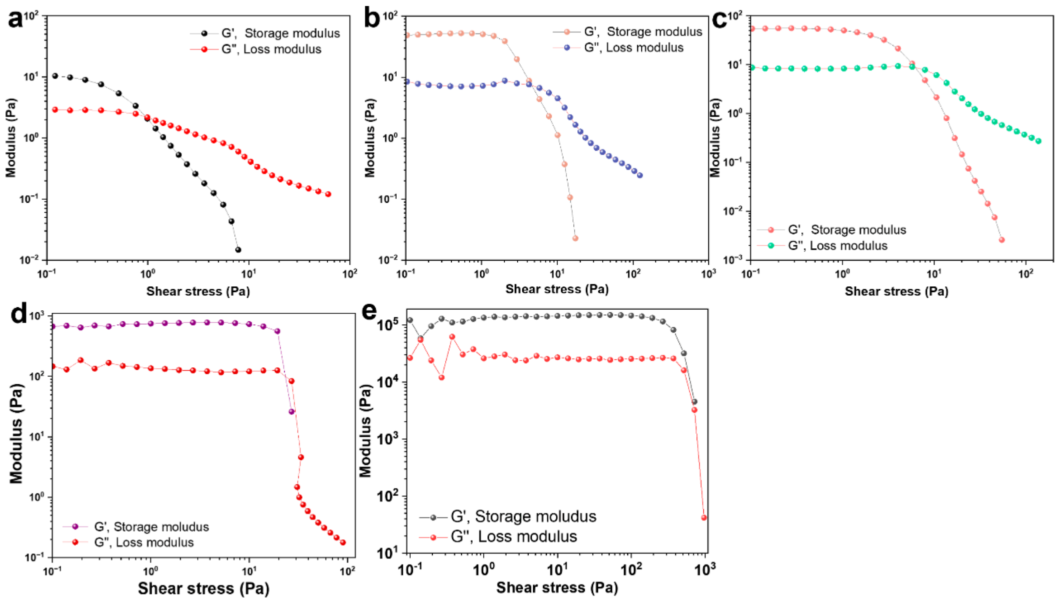

Figure S7. Storage modulus (G') and loss modulus (G'') as a function of shear stress for each MXene@PANI composite ink (the concentrations were (**a**) 150 mg/mL, (**b**) 200 mg/mL, (**c**) 250 mg/mL, (**d**) 300 mg/mL and (**e**) 350 mg/mL respectively).

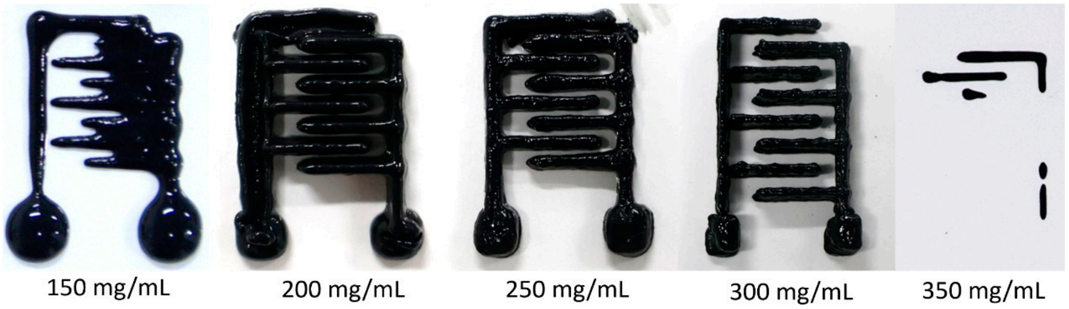

Figure S8. Optical photographs of interdigital electrodes printed with different concentrations of MXene@PANI composite ink.

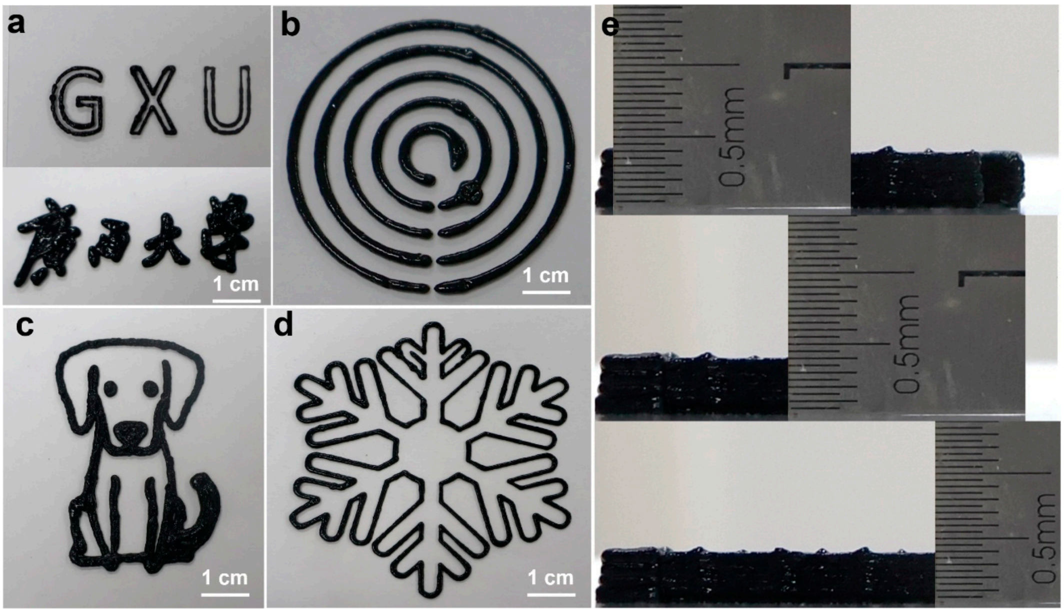

Figure S9. Printing of (**a**) English abbreviation and Chinese characters of Guangxi University, (**b**) concentric circles, (**c**) puppy and (**d**) snowflake patterns with MXene@PANI composite inks. (**e**) Measurement of MXene@PANI electrode thickness.

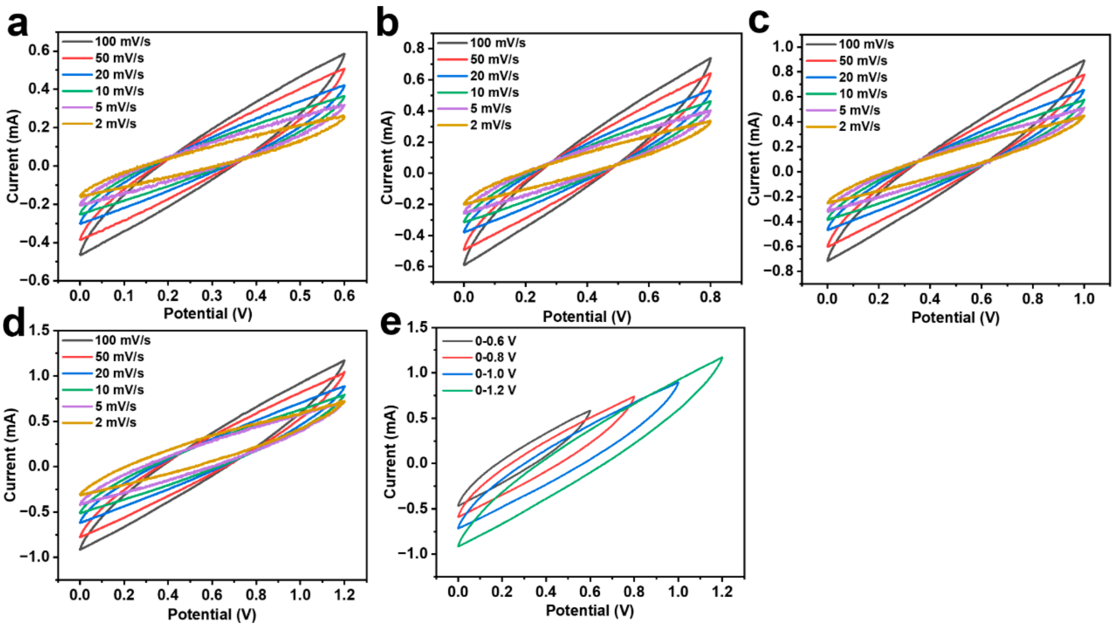

Figure S10. Optimization of the voltage window. (**a**–**d**) CV curves in the 0–0.6 V, 0–0.8 V, 0–1.0 V and 0–1.2 V voltage window. (**e**) Comparison of CV curves for different voltage windows with a scan rate of 100 mV/s.

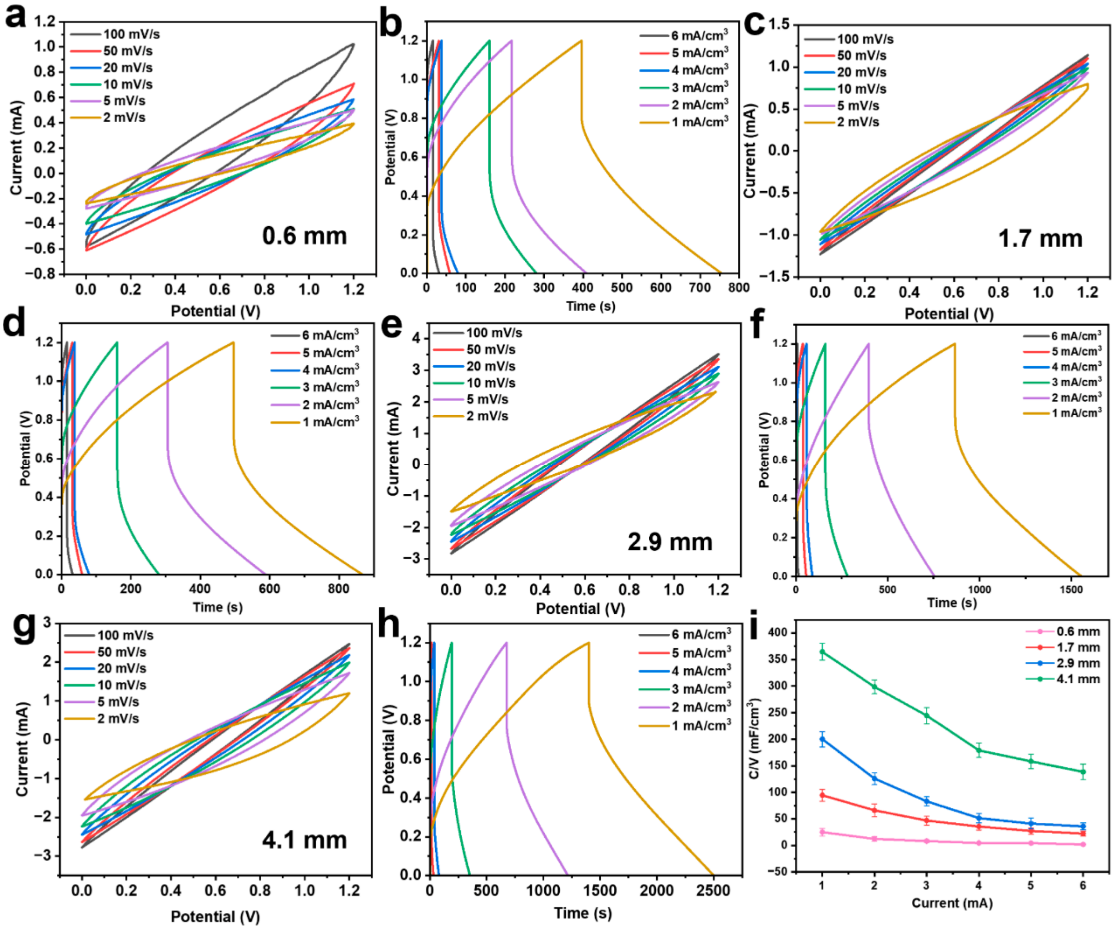

Figure S11. Optimization of the thickness of electrode. (**a**–**b**) CV and GCD curves of 0.6 mm MXene electrode. (**c**–**d**) CV and GCD curves of 1.7 mm MXene electrodes. (**e**–**f**) CV and GCD curves of 2.9 mm MXene electrode. (**g**–**h**) CV and GCD curves of 4.1 mm MXene electrode. (**i**) The volumetric capacitance of MXene MSC of various thickness at different current densities.

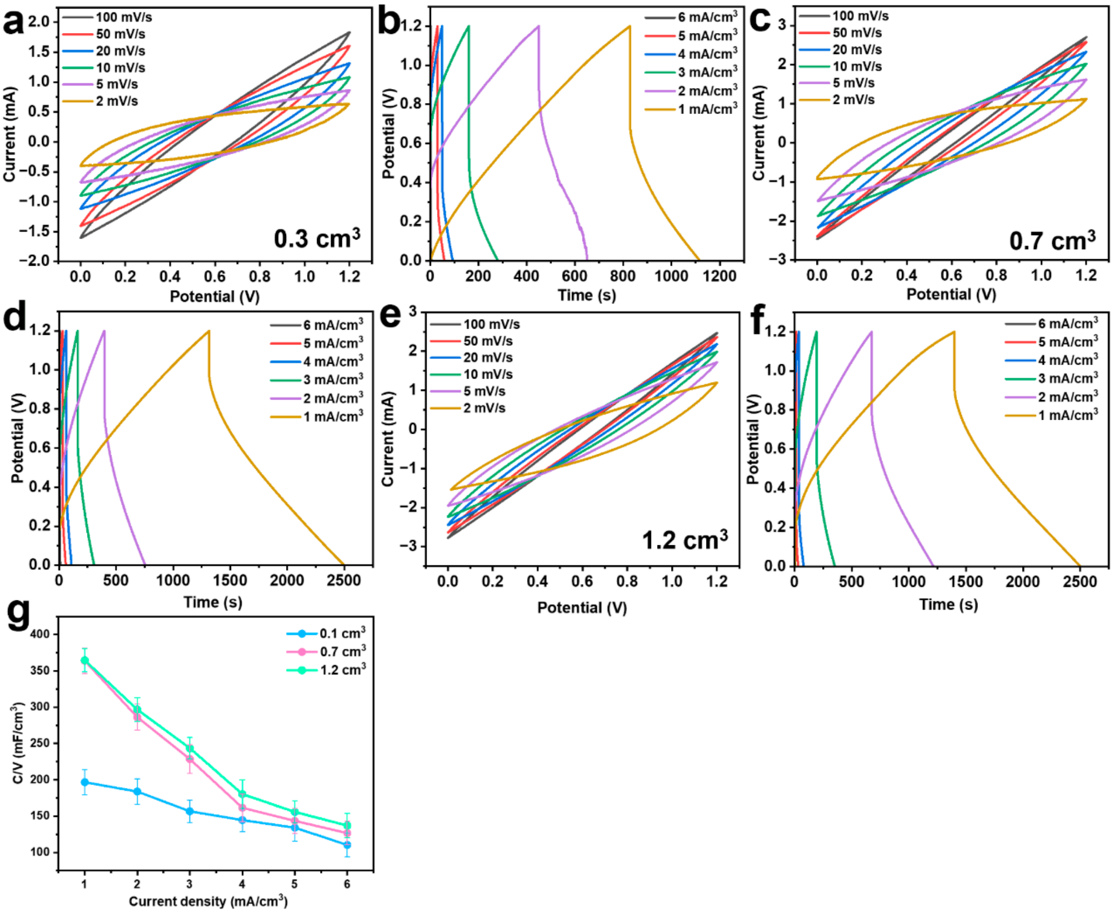

Figure S12. Optimization of electrode sizes. (**a**–**b**) CV and GCD curves of 0.3 cm^3 MXene electrodes. (**c**–**d**) CV and GCD curves of 0.7 cm^3 MXene electrode. (**e**–**f**) CV and GCD curves of 1.2 cm^3 MXene electrode. (**g**) The volumetric capacitance of MXene MSC of various layers at different current densities.

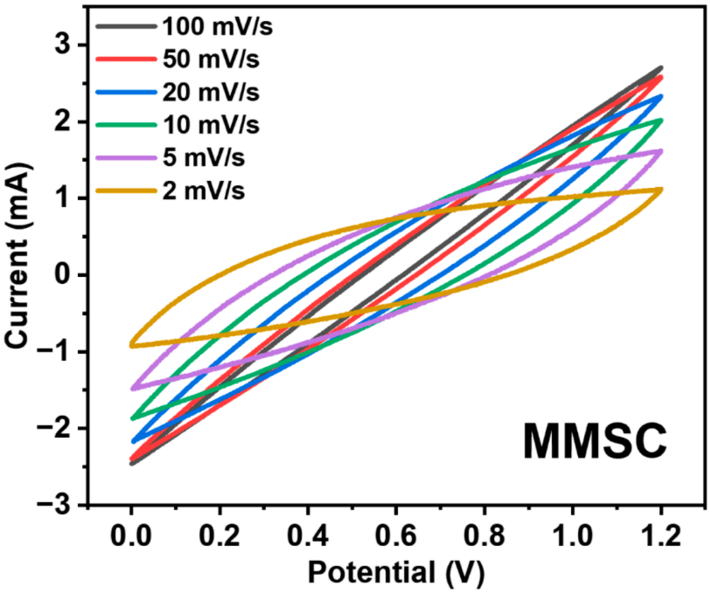

Figure S13. CV curves of MMSC at different scanning rates.

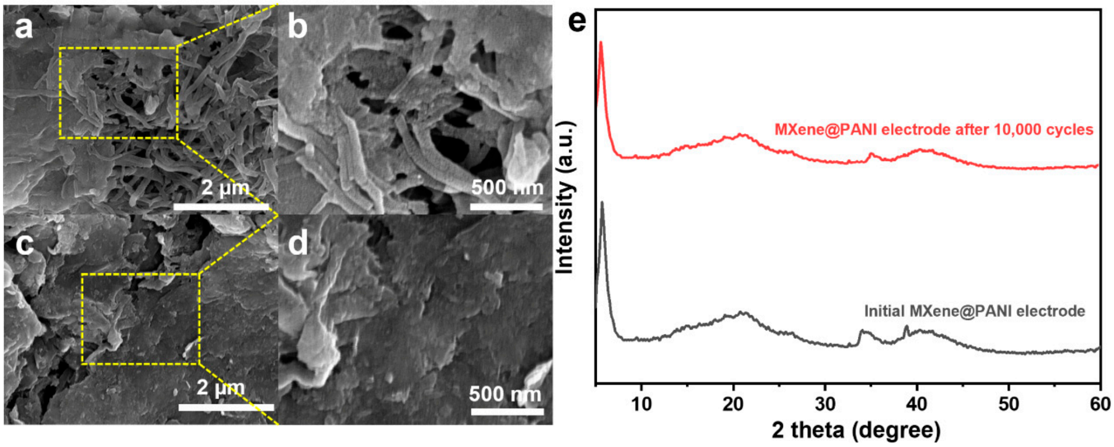

Figure S14. SEM images of (a-b) initial MXene@PANI electrode and (c-d) MXene@PANI electrode after 10000 charging/discharging cycles. (e) XRD pattern of initial MXene@PANI electrode and MXene@PANI electrode after 10000 charging/discharging cycles.

Table S1. Energy density and power density of different devices based on MXene/conductive polymers.

Devices	Structure	Size	Energy density (mWh/cm^3)	Corresponding power density (mW/cm^3)	Ref.
MXene/PANI NPs-10% symmetric SC	PANI NPs interlayered between MXene nanosheets	Diameter 4 cm Thickness 21.6 μm	20.9	88.5	[1]
MXene/PANI //gelatin/poly acrylamide-$Fe_{0.3M}$ SC	MXene/PANI//gelatin/polyacrylamide-$Fe_{0.3M}$	Length 10 mm Width 10 mm Thickness 3.1 mm	0.4	2.172	[2]
PEDOT:PSS/ $Ti_3C_2T_x$ SC	PEDOT:PSS/ $Ti_3C_2T_x$	Length 12 mm Width 6 mm Thickness 795 μm	0.45	22.86	[3]
Ti_3C_2/PPy film-based SC	Ti_3C_2/PPy film	Thickness 12 μm	2.83	70.42	[4]
Ti_3C_2/PEDOT fiber-shaped SC	Ti_3C_2/PEDOT fiber	Length 10 mm Diameter 6 mm	46.96	695.65	[5]
MMSC	Pure MXene	Thickness 4.1 mm Volume 0.7 cm^3	72.4	222.1	This work
MPMSC	2D MXene nanosheets @1D PANI nanofibers	Thickness 4.1 mm Volume 0.7 cm^3	328.2	482.2	This work

References

1. Wang, X.; Wang, Y. M.; Liu, D. D.; Li, X. L.; Xiao, H. H.; Ma, Y.; Xu, M.; Yuan, G. H.; Chen, G. R. Opening MXene Ion Transport Channels by Intercalating PANI Nanoparticles from the Self-Assembly Approach for High Volumetric and Areal Energy Density Supercapacitors. *ACS Appl. Mater. Interfaces* **2021**, *13*, 30633. https://doi.org/10.1021/acsami.1c06934.
2. Han, L.; Li, Y. Q.; Chen, C.; Liu, L. K.; Lu, Z. C. Multifunctional enhanced energy density of flexible wide-temperature supercapacitors based on MXene/PANI conductive hydrogel. *Chem. Eng. J.* **2024**, *485*, 149951. https://doi.org/10.1016/j.cej.2024.149951.
3. Li, L. L.; Meng, J.; Bao, X. R.; Huang, Y. P.; Yan, X.-P.; Qian, H.-L.; Zhang, C.; Liu, T. X. Direct-Ink-Write 3D Printing of Programmable Micro-Supercapacitors from MXene-Regulating Conducting Polymer Inks. *Adv. Energy Mater*. **2023**, *13*, 2203683. https://doi.org/10.1002/aenm.202203683.
4. Zhang, C.; Xu, S. K.; Cai, D.; Cao, J. M.; Wang, L. L.; Han, W. Planar supercapacitor with high areal capacitance based on Ti_3C_2/Polypyrrole composite film. *Electrochim. Acta*. **2020**, *330*, 135277. https://doi.org/10.1016/j.electacta.2019.135277.
5. Wang, M. X.; Chen, Z. W.; Dong, L.; Wu, J. J.; Li, C.; Gao, Q.; Shi, J.; Zhu, C. H.; Morikawa, H. Conductance-stable and integrated helical fiber electrodes toward stretchy energy storage and self-powered sensing utilization. *Chem. Eng. J*. **2023**, *457*, 141164. https://doi.org/10.1016/j.cej.2022.141164.
